# Comprehensive Analysis of Glycolytic Enzymes as Therapeutic Targets in the Treatment of Glioblastoma

**DOI:** 10.1371/journal.pone.0123544

**Published:** 2015-05-01

**Authors:** Morgane Sanzey, Siti Aminah Abdul Rahim, Anais Oudin, Anne Dirkse, Tony Kaoma, Laurent Vallar, Christel Herold-Mende, Rolf Bjerkvig, Anna Golebiewska, Simone P. Niclou

**Affiliations:** 1 NorLux Neuro-Oncology Laboratory, Department of Oncology, Luxembourg Institute of Health (L.I.H.), Luxembourg, Luxembourg; 2 Genomics Research Unit, Luxembourg Institute of Health (L.I.H.), Luxembourg, Luxembourg; 3 Experimental Neurosurgery, Department of Neurosurgery, University of Heidelberg, Heidelberg, Germany; 4 NorLux Neuro-Oncology Laboratory, Department of Biomedicine, University of Bergen, Bergen, Norway; 5 KG Jebsen Brain Tumour Research Center, Department of Biomedicine, University of Bergen, Bergen, Norway; University of Michigan School of Medicine, UNITED STATES

## Abstract

Major efforts have been put in anti-angiogenic treatment for glioblastoma (GBM), an aggressive and highly vascularized brain tumor with dismal prognosis. However clinical outcome with anti-angiogenic agents has been disappointing and tumors quickly develop escape mechanisms. In preclinical GBM models we have recently shown that bevacizumab, a blocking antibody against vascular endothelial growth factor, induces hypoxia in treated tumors, which is accompanied by increased glycolytic activity and tumor invasiveness. Genome-wide transcriptomic analysis of patient derived GBM cells including stem cell lines revealed a strong up-regulation of glycolysis-related genes in response to severe hypoxia. We therefore investigated the importance of glycolytic enzymes in GBM adaptation and survival under hypoxia, both in vitro and in vivo. We found that shRNA-mediated attenuation of glycolytic enzyme expression interfered with GBM growth under normoxic and hypoxic conditions in all cellular models. Using intracranial GBM xenografts we identified seven glycolytic genes whose knockdown led to a dramatic survival benefit in mice. The most drastic effect was observed for *PFKP* (PFK1, +21.8%) and *PDK1* (+20.9%), followed by *PGAM1* and *ENO1* (+14.5% each), *HK2* (+11.8%), *ALDOA* (+10.9%) and *ENO2* (+7.2%). The increase in mouse survival after genetic interference was confirmed using chemical inhibition of PFK1 with clotrimazole. We thus provide a comprehensive analysis on the importance of the glycolytic pathway for GBM growth in vivo and propose PFK1 and PDK1 as the most promising therapeutic targets to address the metabolic escape mechanisms of GBM.

## Introduction

With a prevalence of 2–3 cases per 100,000 people per year in Europe and North America, glioblastoma (GBM) is the most common primary brain tumor and also the deadliest one. The 5-year survival rate remains below 10% and median life expectancy does not exceed fifteen months [1]. Malignancy parameters, such as extensive angiogenesis, hypoxia and necrosis are hallmarks of GBMs that distinguish them from lower grade gliomas. Over the past decade, anti-angiogenic therapy has received considerable attention and a number of clinical trials have been conducted where the treatment was thought to prevent tumor development by inhibiting blood vessel formation and at the same time improve the delivery of chemotherapeutic agents via the functional normalization of existing blood vessels [2]. However, in the clinic, bevacizumab, an antibody targeting vascular endothelial growth factor (VEGF), failed to significantly improve overall patient survival [3, 4] suggesting that tumors quickly develop escape mechanisms [5]. In preclinical studies based on patient-derived GBM xenografts, we showed that bevacizumab reduced tumor blood flow and led to increased invasiveness and hypoxia [6]. Moreover, we observed a significant increase in lactate production within the tumors, and more recently we were able to confirm the induction of a glycolytic switch corresponding to uncoupling glycolysis from oxidative phosphorylation of the tricarboxylic acid (TCA) cycle in response to bevacizumab treatment [6, 7]. Thus the induction of hypoxia and the activation of the glycolytic pathway may mediate glioma resistance to anti-angiogenic treatment, suggesting that targeting the glycolytic pathway may represent a favorable therapeutic approach against GBM [8]. Moreover most solid tumors show increased glycolytic activity independent of oxygen supply (Warburg effect), which favors the production of biomass to sustain tumor cell proliferation through the generation of metabolic intermediates [9]. GBMs in particular display prominent areas of hypoxia surrounded by pseudopalissading cells suggesting that glycolysis is an interesting target for these tumors even at baseline. The glycolytic properties of cancer are demonstrated in the clinic by using positron emission tomography (PET) where the radiolabelled glucose analog, the 2-deoxy-2-(^18^F)fluoro-D-glucose (^18^F-FDG) is avidly taken up by tumor cells, including GBM [10].

Glycolysis represents a ten-step metabolic pathway that implicates more than 15 enzymes. The first glycolytic step generating glucose-6-phosphate is catalyzed by hexokinases (HK), where in particular the HK2 isoform is strongly expressed in cancer including GBM [11]. The phosphorylation of fructose-6-phosphate into fructose-1,6-biphosphate by phosphofructokinase 1 (PFK1), encoded by the *Phosphofructokinase*, *platelet (PFKP)* gene, is the rate limiting step of glycolysis. PFK1 modulation implicates several allosteric regulations including ATP, phosphoenolpyruvate (PEP) and fructose-2,6-biphosphate. From fructose-1,6-biphosphate, the sequential activities of aldolase A (ALDOA), glyceraldehyde 3-phosphate dehydrogenase (GAPDH) and phosphoglycerate kinase 1 (PGK1) lead to the generation of 3-phosphoglycerate, which is isomerized to 2-phosphoglycerate by phosphoglycerate mutase 1 (PGAM1). The latter was found to be over-expressed in glioma and to correlate with glioma grade [12]. Among the three enolase isoforms (ENO1-3) generating PEP, only ENO1 and ENO2 are expressed in the brain, and ENO1 is the major isoform in GBM (75–90% of total cellular enolase activity) [13]. A key glycolytic enzyme which is consistently altered in cancer cells is pyruvate kinase the enzyme catalyzing the conversion of PEP to pyruvate and ATP [14]. The fetal M2 isoform (PKM2) is more abundant in cancer cells and favors aerobic glycolysis to the benefit of anabolic processes in highly proliferating cells [15]. Finally, pyruvate dehydrogenase kinase 1 (PDK1), which is strongly expressed in GBM compared to normal brain [16], inhibits pyruvate dehydrogenase, thereby preventing the entry of pyruvate into the TCA cycle.

Here, we address in a comprehensive manner the role of glycolytic induction in hypoxic GBM cells and provide evidence through genetic and chemical interference that inhibition of the glycolytic pathway strongly affects GBM growth in patient-derived intracranial mouse models. Using transcriptomic and functional knockdown strategies in vitro and in vivo, we identify PFK1, PDK1, PGAM1, ENO1, HK2, ALDOA and ENO2 as key glycolytic enzymes essential for GBM growth and propose them as potential therapeutic targets against GBM.

## Materials and Methods

### Ethics statement

Human glioblastoma biopsies were obtained from the Neurosurgery Department of the Centre Hospitalier in Luxembourg (CHL) (T16) or the Department of Neurosurgery, Haukeland University Hospital in Bergen (P3, P8), Norway. All patients had provided written informed consent, with procedures that were approved for the project (project number: REC-LRNO-20110708) by the National Research Ethics Committee for Luxembourg (CNER) or by the Regional Ethical Board at the Haukeland University Hospital in Bergen. The handling of the animals and the surgical procedures were performed in accordance with the European Directive on animal experimentation (2010/63/EU) and the national regulations of Luxembourg and the local ethical committee (the Animal Welfare Structure (AWS) LIH) approved the protocol.

### Cell culture

Five patient-derived glioblastoma stem-like cell lines NCH421k, NCH660h, NCH465, NCH601 and NCH644 were generated in the laboratory of Dr Christel Herold-Mende (Department of Neurosurgery, University of Heidelberg) [17]. NCH421k, NCH660h, NCH465, NCH601 were cultured as non-adherent spheres in DMEM-F12 medium (Lonza) containing 1xBIT100 (Provitro), 2mM L-Glutamine, 30U/ml Pen-Strep, 1U/ml Heparin (Sigma), 20ng/ml bFGF (Miltenyi, 130-093-841) and 20ng/ml EGF (Provitro, 1325950500). NCH644 were grown in Neurobasal base medium (Life Technologies) supplemented with 1xB-27 (Life Technologies) 2mM L-Glutamine, 30U/ml Pen-Strep, 1U/ml Heparin (Sigma), 20ng/ml bFGF (Miltenyi, 130-093-841) and 20ng/ml EGF (Provitro, 1325950500). U251 cells were kindly provided by Dr. J. Carlsson, Uppsala University, Sweden [18]. U87 and T98G cells were obtained from the ATCC (Rockville, USA). U87 U251 and T98G cells were cultured as adherent monolayers in DMEM containing 10% FBS, 2mM L-Glutamine and 100U/ml Pen-Strep (all from Lonza). Normal human astrocytes (NHA), kindly provided by Dr Uros Rajcevic (National Institute of Biology, Ljubljana, Slovenia) were acquired from Lonza. NHA were grown in DMEM, 20% FBS, 2mM L-Glutamine and 100U/ml Pen-Strep (all from Lonza). Normoxic cultures were kept at 37°C under 5% CO_2_ atmospheric oxygen. Hypoxic conditions at 0.1% O_2_ were maintained in a hypoxic incubator chamber (Galaxy 48R incubator, New Brunswick, Canada).

### Organotypic GBM biopsy derived spheroids

Organotypic GBM spheroids from patient samples were prepared as previously described [6, 19] and maintained in spheroid medium (DMEM medium, 10% FBS, 2mM L-Glutamine, 0.4mM NEAA and 100U/ml Pen-Strep; all from Lonza) in agar pre-coated flasks for 7–10 days. Primary adherent P3 cell line (P3A) was derived by plating P3 spheroids in uncoated flasks in spheroid medium until a confluent culture was obtained.

### Genome-wide expression analysis

Total RNA was extracted using QIAGEN RNeasy Mini Kit (Qiagen), according to the manufacturer’s protocol. GeneChip Human Gene 1.0ST Arrays (Affymetrix) were used to determine the genome-wide expression profiles. Total RNAs (250ng) were processed using the Affymetrix WT Expression kit before being hybridized on Affymetrix GeneChip Human Gene 1.0 ST arrays according to the manufacturer’s instructions (protocol P/N 702808 Rev.6). Upon hybridization, microarrays were washed, stained and scanned according to manufacturer’s standard procedures. CEL files containing hybridization raw signal intensities were imported into the Partek GS software for further statistical analysis. First, probe intensities were summarized to gene expression signals using Partek default options (GC-content adjustment, RMA background correction, quantile normalization, log2 transformation and summarization by means. Gene expression profiles were established for cells cultured in normoxia and severe hypoxia (0.1% O_2_) at two time points (12hours = 12h and 7 days = 7d). Lists of differentially expressed genes (DEGs) were obtained with ANOVA (False discovery rate (FDR) <0.001, any fold change (FC)). P-values were adjusted by Benjamin and Hochberg’s False Discovery Rate (FDR) [20]. The Ingenuity Pathway Analysis (IPA, Ingenuity Systems) and the DAVID database (DAVID 6.7; http://david.abcc.ncifcrf.gov/) [21] were used for data mining. Right-tailed Fisher’s exact test was used to calculate a p value for functional enrichment analysis in the IPA (threshold:-log(p-value)> 1,3). REVIGO server was used for summarization of the altered Gene Ontology (GO) terms [22]. Venn diagram analysis was performed with the SAB lab web tool (http://sablab.net/venn.php). Microarray data are available in the ArrayExpress database (www.ebi.ac.uk/arrayexpress) under accession number E-MTAB-3085.

### Quantitative real-time PCR (QPCR)

1μg of total RNA was extracted using Qiagen RNeasy Mini Kit (Qiagen) and reverse transcribed to cDNA using the iScript cDNA Synthesis Kit (Bio-Rad), according to the manufacturer’s protocol. QPCR was carried out using Fast SYBR Green Master Mix and the Viia 7 Real Time PCR System (Life Technologies; Ta = 60°C QPCR reaction was performed in 5μL volumeFold-change (FC) was calculated using the ΔΔC_t_ method and normalized to the expression of *EZRIN* (QBase software). See S1 Table for list of primers used.

### Gene knockdown

Gene knockdown of glycolytic genes was performed in NCH644, NCH421k and U87 using lentiviral particles expressing specific shRNAs (shRNA sequences in S2 Table). Individual pGIPZ shRNAmir (Open Biosystems) constructs were obtained as *E*. *coli* cultures in LB-lenox (low salt) medium with 8% glycerol, 100 μg/ml carbenicillin and 25 μg/ml zeocin. Plasmids were amplified and purified using the Qiagen Plasmid Midi kit (Qiagen). Lentiviral particles were produced in HEK cells by co-transfection of the pGIPZ-shRNAmir-control (Scramble) or pGIPZ-shRNAmir-target gene vector with the viral core packaging construct pCMVdeltaR8.74 and the VSV-G envelope protein vector pMD.G.2 as previously described [23]. Supernatant containing viral particles was used to transduce 100,000 glioma cells and puromycine selection (0.5ug/mL for NCH421k, 1ug/mL for NCH644, 5ug/mL for U87) was applied to obtain stably transduced GFP-positive cells.

### 3D-sphere assay

96-well plates were pre-coated with 1.5% Noble Agar (BD Biosciences) to prevent cell attachment. 1,000 NCH644 or U87 cells were seeded in each well and the plates were incubated at 37°C, 5% CO_2_ with gentle agitation to obtain uniform 3D spheres. Spheres were cultured for 7 days under severe hypoxia (0.1% O_2_) in their respective media.

### Cytotoxicity assay

Cytotoxicity test was carried out for clotrimazole (range 5–75μM), citrate (range 5-30mM), sodium dichloroacetate (DCA, range 0.5-50mM) and 3-bromopyruvate (BPA, range 50–700 μM) (all from Sigma). Adherent cells were plated in semi-confluent concentration in 96 well plates. Increasing concentrations of compounds or DMSO were applied for 72h. Induction of cell death was measured with the Sulforhodamine (SRB) assay (In Vitro Toxicology Assay Kit, Sulforhodamine B based, Sigma) according to the manufacture’s protocol. The optical density was measured at 540nm. The percentage inhibition of cell mass was determined as: % reduction of cell mass = (Mean OD_control—_Mean OD_sample_) X 100/ Mean OD_control_. IC_50_ was determined with the GraphPad Prism 5 software.

To uniform the primary cultures for repeatable drug testing we created 3D spheroids form equal amount of tumor cells sorted directly from the xenografts samples. Using eGFP expressing mice for generation of spheroid-based xenografts [24] allowed us to create uniform spheroids composed of tumor cells only. 1000 tumor cells per well were plated in 96 well plates pre-coated with agar. Spheroids were used 3 days after sort and the size remained relatively similar within the first 7 days of culture in normoxia and hypoxia. As control NHA cells were also cultured under non-adherent conditions. Treatment was carried out for 72h in normoxic and hypoxic (0.1% O_2_) conditions with 30μM clotrimazole (Sigma) and spheroid viability was tested with cell viability test. Experiments were carried out with at least 5 spheroids (n = 3).

### Cell viability test

Cell viability in 3D cultures was assessed with the LIVE/DEAD Viability/Cytotoxicity assay kit (Molecular Probes). Spheres and spheroids were stained for 6 hours and measurements of viable (Calcein, ‘green’ florescence) and dead (Ethidium homodimer-1, ‘red’ fluorescence) cells were performed using fluorescence confocal microscopy (Zeiss LSM STO META) by obtaining 20–25 stacks of two-dimensional images from successive focal planes (5 μm). Quantification was performed using IMARIS software. The volume of viable and dead cells was calculated by multiplying the surface area of each component (‘green’ or ‘red’) per stack by the total height of the image stacks. The percentage of dead cell volume was calculated as: % dead cell in spheroids (volume) = Dead cell volume (‘red’) x 100/Total spheroid volume (‘green’ + ‘red’).

### Lactate release assay

Cells were incubated for 48h in normoxia or 0.1% O_2_ hypoxia. The culture medium was removed and filtered with the 10kD spin filter. The flow-through was diluted 20 times before the assay. Lactate concentration was established with the Lactate Assay Kit (BioVision) according to the manufacture’s protocol and measured with the FLUORstar Optima (BMG Labtech). Results were normalized against the total number of cells in each sample.

### In vivo shRNA screen

55 NCH421k cell lines harboring different shRNA knockdown and NCH421k cells with a control vector were premixed before implantation (55 x 2’500 = 137’500 cells). The total pool was implanted intracranially to immunodeficient NOD/SCID mice (n = 5). Mice were anesthetized with a mixture of ketamine (100mg/kg) and xylazine (10mg/kg) and fixed in a stereotactic frame (Narishige Group, Tokyo, Japan). Tumor cells (137’500 cells) were implanted into the right frontal cortex using a Hamilton syringe (Hamilton, Reno, NV, USA) and analgesic (Buprenorphine, 0.1 mg/kg) was administered subcutaneously to relieve post-operative pain. Mice were fed a standard pellet diet and were provided with water ad libitum. Animals were euthanized by cervical dislocation at the appearance of neurological symptoms and weight loss. Brains were removed and the presence of a tumor was assessed by *ex vivo* IVIS imaging (IVIS Lumina Fluorescence system; PerkinElmer Compagny). As a control 55 pooled cell lines was cultured for the same period of time (n = 3). Total DNA was extracted from pooled cells before implantation (‘Baseline level’), from developed tumor tissue and in vitro cultures. Barcodes were amplified by PCR with specific primers: Forward (5’-CAAGGGGCTACTTTAGGAGCAATTATCTTG-3’), Reverse (5'-GGTTGATTGTTCCAGACGCGT-3') and 300ng of DNA template PCR reaction parameters: 94°C for 3min followed by 94°C for 35s, 62°C for 35s and 72°C for 1min (30 cycles). The quality of PCR amplification was assessed by the migration on 1.5% agarose gel (expected size: 250bp). The abundance of each shRNA was quantified by next generation sequencing (NGS Junior, Roche). Data were processed using Fuzznuc (http://emboss.sourceforge.net/apps/cvs/emboss/apps/fuzznuc.html). Depletion of the essential genes was calculated by comparing results from in vivo (n = 5) and in vitro control screen (n = 3) or DNA isolated directly after pooling (‘Baseline level’ for *PGAM1* and *PFKP*).

### Survival study

NCH421k cell clones harboring a gene specific shRNA were stereotactically implanted in immunodeficient NOD/SCID mice (6–7 mice/clone, NOD.CB17-Prkdc scid/J, Charles River, Lyon). Control mice were implanted with cells carrying a scramble shRNA construct (21 mice). Mice were anesthetized with a mixture of ketamine (100mg/kg) and xylazine (10mg/kg) and fixed in a stereotactic frame (Narishige Group, Tokyo, Japan). Tumor cells (137’500 cells) were implanted into the right frontal cortex using a Hamilton syringe (Hamilton, Reno, NV, USA). Four parameters were monitored daily to evaluate brain tumor severity as described previously [19]: weight (loss superior to 10% of the starting weight), coat, lordosis and CNS symptoms, using a score between 0 (no symptoms) and 3 (severe symptoms). The animals were euthanized by cervical dislocation when one criterion reached 3 or when 3 criteria reached 2. After sacrifice, in situ fluorescent images of brain tumors were generated using the IVIS instrument. Half of each brain was embedded in paraffin for histology and half was frozen for knockdown efficiency evaluation by QPCR.

For clotrimazole treatment, GBM patient-derived P3 spheroids (6 spheroids/mice) were stereotactically implanted in the brain of nude mice (Crl:NU(Ico)-Foxn1nu, Charles River, Lyon). To facilitate tumor monitoring in vivo P3 cells were transduced with DsRed expressing lentiviral vector. At 3 weeks, the first fluorescent signal was visible (IVIS instrument) and mice were randomly divided in two groups (n = 7): (1) control group receiving saline solution and (2) treated group receiving 3 times weekly clotrimazole (150mg/kg) administered by oral gavage.

## Results

### Glycolysis is the major biological pathway induced by hypoxia

**Fig 1:**
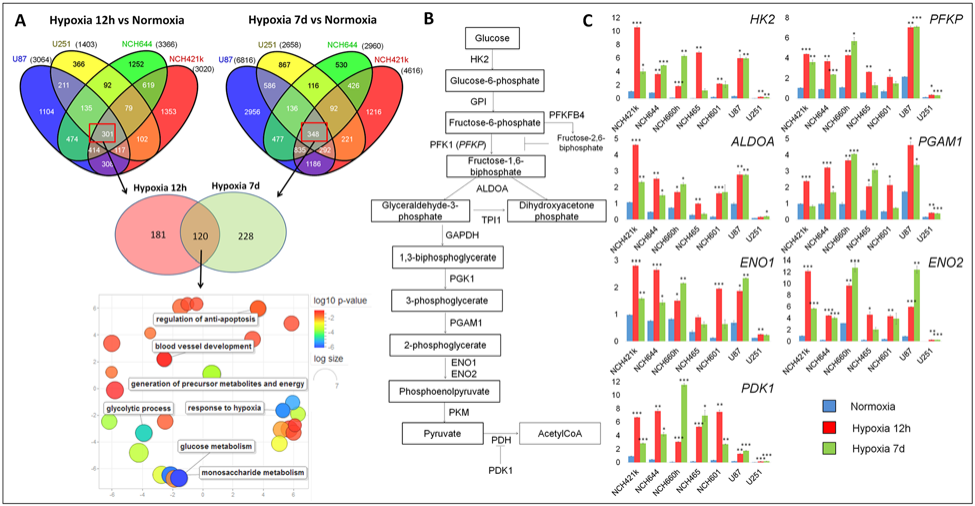
Glycolysis-related genes are up-regulated in glioblastoma cells under hypoxia. **A**. Stem-like (NCH644, NCH421k) and classical adherent (U87, U251) glioma cells were cultured in 0.1% O_2_ for short term (12 hours = 12h) and long term (7 days = 7d). Differentially expressed genes (DEGs) were established between hypoxic and normoxic cells (n = 3–6). Venn diagrams (top) represent analysis of DEGs after 12h and 7d respectively (FDR<0.001; any fold change (FC)). Red squares highlight the genes commonly modulated in all four glioma cell lines. 120 genes were commonly deregulated upon 12h and 7d hypoxia (Venn diagram, middle) which were strongly associated with glycolysis (9 genes) and glucose metabolism (11 genes) (Revigo representation of significant GO terms, bottom). **B**. Schematic representation of the glycolytic pathway and associated enzymes. HK2 = hexokinase 2; PFK1 = phosphofructokinase 1 (encoded by *PFKP* = *Phosphofructokinase*, *platelet*); ALDOA = aldolase A; PGAM1 = phosphoglycerate mutase 1; ENO1 = enolase 1; ENO2 = enolase 2; PDH = pyruvate dehydrogenase; PDK1 = pyruvate dehydrogenase kinase 1. **C**. Quantitative PCR analysis of glycolytic gene expression in adherent glioma cells (U87 and U251) and glioma stem-like cells (NCH421k, NCH644, NCH660h, NCH465 and NCH601), under normoxia and hypoxia (12h and 7d). Data are presented as mean +/- SEM (n = 3). Data were normalized against *EZRIN* expression. NCH421k cells were used as an internal calibration (value = ‘1’); * p<0.05; p**<0.01; p***<0.001.

A drastic effect of hypoxia on gene expression has been widely demonstrated in several cancer types [25], however, the activated pathways in response to oxygen deprivation are largely cell type dependent [26]. To determine the response of GBM to hypoxia, we performed a genome-wide transcriptomic analysis on glioma stem-like cells (NCH644 and NCH421k) and on classical adherent glioma cells (U87 and U251) under short (12h) and long term (7d) severe hypoxia (0.1% O_2_). The expression profiles were determined for each glioma cell line and the lists of differentially expressed genes (DEGs) were established by comparing hypoxia (12h or 7d) with normoxia (2-way ANOVA, FDR<0.001, any fold change). We further extracted genes commonly altered in all cells analysed (Fig 1A top). Interestingly, the number of common DEGs was similar after 12h and 7d (301 and 348 genes respectively). Using the DAVID functional annotation tool [27, 28], we found that glucose metabolism, and more specifically glycolysis, was highly enriched under hypoxia in both conditions (S1A Fig). This was confirmed by Ingenuity Pathway Analysis (IPA] where glycolysis was shown to be significantly altered in all hypoxic GBM cells (-log(p-value] = 7.28 and 7.75 upon 12h and 7d hypoxia respectively]. The comparison of DEGs after 12h and 7 days in hypoxia revealed that 120 genes were deregulated in both conditions (Fig 1A middle). Next to glycolysis and glucose metabolism the commonly deregulated genes were associated also to pathways regulating general hypoxia response, metabolism, apoptosis and angiogenesis (Fig 1A bottom).

To further select the genes most upregulated upon hypoxia we have imported the list of glycolysis-associated genes from Gene Onthology (GO:0006096) and KEGG databases (hsa00010, glycolysis/gluconeogenesis, [29]) and analysed gene expression changes occurring upon hypoxia (S3 Table). Importantly, the majority of core glycolytic genes (Fig 1B, S3 Table) were up-regulated whereas the pentose phosphate pathway (PPP)-related genes were down-regulated (S3 Table). This is in agreement with recent data showing that PPP enzymes are reduced in response to acute hypoxia, but their expression is up-regulated in vivo after prolonged hypoxia [7, 30].

We selected seven glycolytic genes (*HK2*, *PFKP*, *ALDOA*, *PGAM1*, *ENO1*, *ENO2* and *PDK1*; Fig 1B) and confirmed their strong up-regulation under hypoxia by QPCR in seven GBM cell lines (five patient derived glioma stem-like cells and two adherent GBM cell lines; Fig 1C). The increase of glycolytic genes was observed for all glioma cells already after 12h hypoxia and remained activated up to 7 days. Interestingly, in many cases the response was stronger in short term hypoxia, while expression levels had slightly decreased by 1 week. Also, the basal expression level of glycolytic genes and their induction by hypoxia was lower in U251 compared with other glioma cells.

To confirm the upregulation of glycolysis at the functional level we monitored the release of lactate in the extracellular medium. Under hypoxia, the lactate concentration in the medium was strongly augmented, indicating an increased pyruvate to lactate conversion in all cells, including astrocytes (S1B Fig).

In summary, we demonstrate, using several cellular GBM models, a strong induction of glycolysis under hypoxia, at the level of enzyme expression and functional activity. This metabolic activation highlights the importance of the glycolytic pathway for hypoxic glioma cells.

### Knockdown of glycolytic enzymes affects GBM cell growth under hypoxia

**Fig 2:**
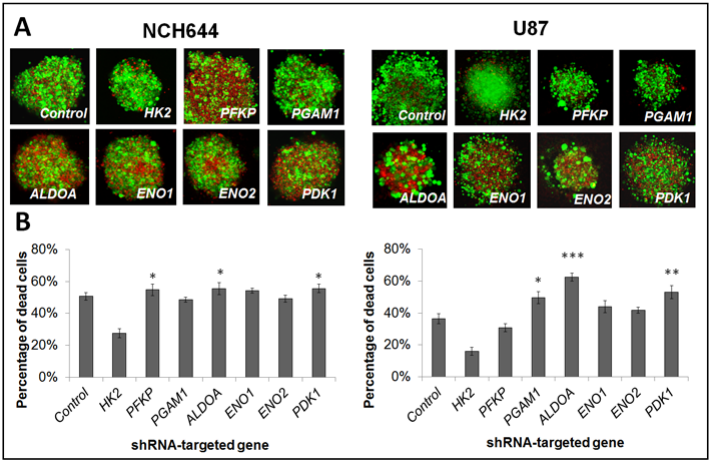
In vitro effect of glycolytic gene knockdown in glioblastoma cells. **A**. Cell viability test of 3D spheres carrying gene knockdowns under long-term (7d) hypoxia. Viable cells = ‘green’, dead cells = ‘red’. Representative images are shown (n = 10). **B**. Quantification of the percentage of dead cells within 3D spheres in hypoxia (n = 10; mean ± SEM) (* p<0.05; ** p<0.01; *** p<0.001).

To address the effect of gene knockdown of glycolytic enzymes on cell survival under hypoxia we used a cell viability assay. We have chosen seven genes that were strongly upregulated under hypoxia in all tumor cells analysed (S3 Table). Knock down efficiency for *HK2*, *PFKP*, *ALDOA*, *PGAM1*, *ENO1*, *ENO2* and *PDK1* in NCH644 and U87 cells is shown in S2 Fig. Since NCH644 cells grow as three dimensional spheres, we also generated spheres from U87 cells. After 7 days in severe hypoxia (0.1% O_2_), spheres were generally smaller compared to those grown under normoxia (not shown), suggesting a lower proliferation rate under hypoxia. To determine the ratio of cell death, cells were stained with calcein (viable cells in green) and with ethidium bromide (dead cells in red) (Fig 2). We observed an increase in the number of dead cells for NCH644 when *PFKP*, *ALDOA* and *PDK1* genes were repressed. Similarly increased cell death was observed in U87 cells after silencing of *ALDOA*, *PDK1* and *PGAM1* (Fig 2A and 2B). Cell viability was not affected in 3D spheres grown in aerobic conditions upon respective gene knockdown (not shown). In summary, these observations indicate that the silencing of glycolytic genes affects cell survival under hypoxia in vitro, however the effect is to some extent enzyme and cell type dependent.

### Glycolytic enzymes are essential for in vivo tumor growth and their inhibition increases mouse survival

**Fig 3:**
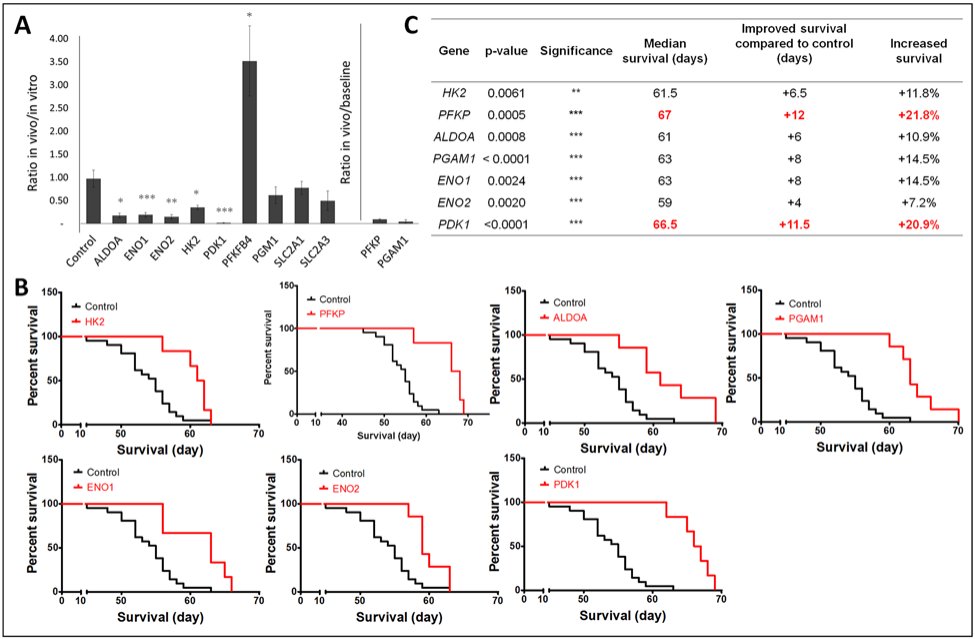
Mouse survival study revealed key glycolysis-related genes for in vivo tumor growth. **A**. Targeted in vivo shRNA screen in NCH421k cells. From 11 glycolytic target genes, five shRNA containing clones were depleted after in vivo growth compared to in vitro culture (*ALDOA*, *ENO1*, *ENO2*, *HK2*, *PDK1*) (* p<0.05; ** p<0.01; *** p<0.001; n = 3 for in vitro, n = 5 for in vivo). The number of shRNAs in each sample was quantified using NGS and is indicated as percentage of control. As *PGAM1* and *PFKP* knockdown clones were strongly depleted both in vivo and in vitro, these results were compared to baseline (original cell pool n = 1, p values not available). **B**. NCH421k cells with the indicated gene specific shRNAs were implanted intracranially into nude mice (n = 21 for control and n = 6–7 for glycolytic genes). Kaplan-Meier graphs show the effect of glycolytic gene knockdown on mouse survival. C. Table summarizing the effect of glycolytic gene knockdown on mouse survival (* p<0.05; ** p<0.01; *** p<0.001).

To determine the effect of glycolytic enzyme silencing on GBM growth in vivo, in the brain of NOD/SCID mice we implanted a pool of 55 shRNA-expressing glioma stem-like cells (NCH421k), targeting a number of different pathways, of which eleven were glycolytic genes found to be differentially expressed under hypoxia (S3 Table). These included the seven genes found to be essential for survival in vitro upon hypoxia *(HK2*, *PFKP*, *PGAM1*, *ALDOA*, *ENO1*, *ENO2* and *PDK1*) and four additional genes upregulated in hypoxia (*PGM1*, *PFKFB4*, *SLC2A1*, *SLC2A3*). A scramble shRNA vector was also included. We assumed that if the gene carried a survival advantage the knockdown cells would be depleted in the growing tumor. This allowed to determine in a single experiment which glycolytic enzymes were relevant for tumor growth in vivo. As a control pooled cells were cultured in vitro for the same period of time. Seven weeks after implantation, mice showed the first neurological symptoms and were sacrificed. Total DNA was extracted from GBM xenografts and in vitro cultures and the number of shRNA molecules was quantified. Interestingly, from the initial eleven shRNA-targeted glycolytic genes, seven (*HK2*, *PFKP*, *PGAM1*, *ALDOA*, *ENO1*, *ENO2* and *PDK1*) were significantly depleted in the xenografts, indicating that cells lacking these genes had a growth disadvantage in vivo (Fig 3A). Since *PGAM1* and *PFKP* were also depleted in the in vitro cultures, we compared their in vivo depletion to baseline (control cell pool before culture) (Fig 3A). *SLC2A3*, *SLC2A1* and *PGM1* were not depleted in vivo, while *PFKFB4* was depleted in vitro but not in vivo.

To determine the effect of knockdown on mouse survival, we separately implanted NCH421k cells expressing each a gene specific shRNA. In confirmation of the previous experiment, seven knockdowns led to a significant increase in mouse survival (Fig 3B and 3C). The most dramatic survival benefit was obtained with *PFKP* (+21.8%) and *PDK1* knockdown (+20.9%). A strong survival effect was also observed after silencing of *PGAM1* and *ENO1* (+14.5%), as well as after *HK2*, *ALDOA*, and *ENO2* knockdown (11.8%, 10.9% and 7.2% respectively) (Fig 3B and 3C). Gene knockdown in the xenografts was confirmed by QPCR on total RNA extracted from frozen tumor tissue, which reached more than 50% for all genes except *ENO1* (S3 Fig). In summary, we provide strong evidence that genetic interference with glycolysis slows GBM growth in vivo and we propose in particular PDK1 and PFK1 (*PFKP*) as promising targets for GBM treatment.

### Increased mouse survival after chemical inhibition of PFK1

**Fig 4:**
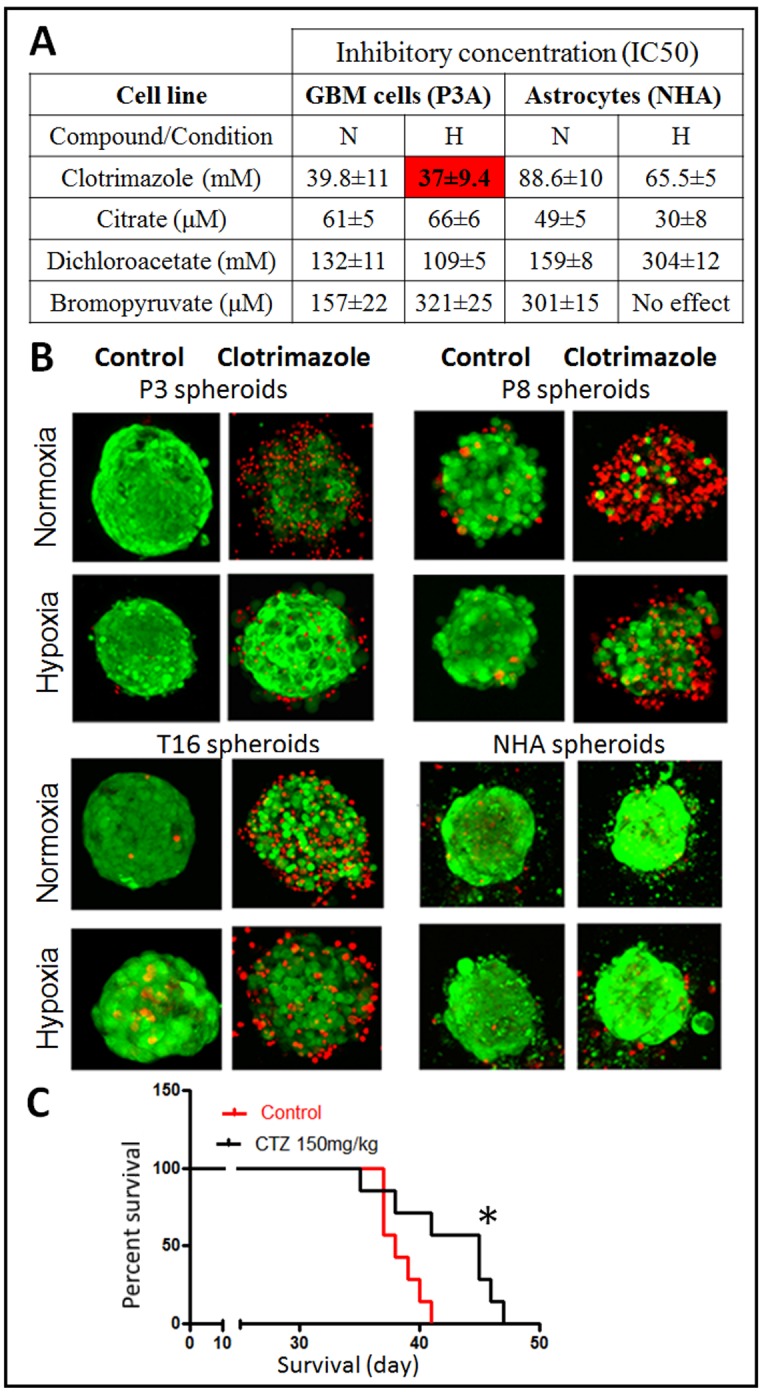
Glycolysis inhibition with clotrimazole affects glioma cell survival in vitro and delays tumor growth in vivo. **A**. The IC_50_ of different glycolysis inhibitors was determined for patient derived GBM cells (P3A) and normal human astrocytes (NHA). N: normoxia, H: hypoxia (0.1% O2). Cells were exposed to indicated compounds for 72h and IC_50_ was determined with the SRB assay (n = 3). **B**. The cytotoxic effect of clotrimazole (30μM) was assessed on organotypic spheroids of several patient-derived GBM (P3, P8, T16) and NHA, treated for 72h in normoxia and 0,1% O_2_ (n = 5). Representative images showing viable cells in ‘green’, dead cells in ‘red’ fluorescence. **C**. P3 spheroids were implanted intracranially and clotrimazole (CTZ, 150mg/kg) treatment was started 3 weeks after implantation (n = 7). Kaplan-Meier curve shows significantly improved mouse survival (* p<0.05).

To determine whether chemical inhibition of glycolysis could be used to slow GBM growth, we tested several compounds previously reported to inhibit glycolytic enzymes. PFK1 can be targeted by clotrimazole and citrate [31, 32], dichloroacetate (DCA) is reported to inhibit PDK activity, whereas 3-bromopyruvate (BPA) is thought to affect HK2 and GAPDH activities [33]. We determined the IC50 of clotrimazole, citrate, DCA and BPA in patient-derived GBM cells (P3A) and normal astrocytes, under both normoxia and hypoxia (Fig 4A). The IC50 was also determined for classical adherent glioma cells U87, U251 and T98G (S4 Table). Except for citrate, GBM cells were more sensitive to drugs than astrocytes and for clotrimazole and DCA the sensitivity was increased under hypoxia. DCA and BPA were generally active only in the high micromolar range. Based on the best efficacy of clotrimazole and the strong impact of *PFKP* knockdown observed in vivo (Fig 3B), we focused on the chemical inhibition of PFK1 by clotrimazole. We evaluated the effect of clotrimazole in several patient-derived organotypic spheroids by performing viabilitytests in vitro. Primary spheroid cultures are more relevant tumor models for drug testing because they maintain the genetic features and some of the tissue complexity of patient tumor [34, 35]. Here we found that, under normoxia, clotrimazole induced cell death in P3, P8 and T16 GBM spheroids but had no effect on astrocyte spheres at the indicated concentration. Similar results were observed under hypoxic conditions (Fig 4B). Based on these data, we determined the effect of clotrimazole on the survival of mice implanted intracranially with a patient-derived GBM spheroids (P3). Clotrimazole (150mg/kg, 3-weekly) led to a small but significant improvement in mouse survival (+7 days; p = 0.0272, Fig 4C), indicating that glycolysis inhibition via chemical interference is effective in the treatment of patient derived GBMs and that PFK1 is a promising target for GBM therapy.

## Discussion

In this study, we undertook a comprehensive analysis of transcriptomic data from patient-derived GBM stem-like cells and classical adherent GBM cell lines grown under short and long-term hypoxia. Using data mining tools, we identified glycolysis as a major pathway enabling the adaptation to oxygen deprivation in all cell lines. The activation of glycolysis was confirmed in all GBM cells studied at the protein level and at the functional level. These findings support the notion that GBM cells heavily rely on metabolic alterations to adapt to a changing microenvironment induced e.g. by anti-angiogenic treatment. By combining a targeted in vivo shRNA screen followed by survival studies in patient derived xenograft mouse models, we uncovered the importance of several key glycolytic enzymes including PFK1, PDK1, PGAM 1 and ENO1 for intracranial growth of GBM. Although to a lesser extent also HK2, ALDOA and ENO2 were identified as important players for tumor progression in vivo. In view of the pronounced hypoxic areas in GBM and the general induction of hypoxia upon anti-angiogenic treatment, these data identify the key targets in the glycolytic pathway relevant for single agent or combination therapies.

The most prominent survival increase was seen after PFKP silencing (+21.8%), providing the first evidence that PFK1 constitutes an important target for GBM therapy. In agreement with this, a recent study identified 6-phosphofructo-2-kinase/fructose-2,6-biphosphatase 4 (PFKFB4) as essential for GBM cell survival in vitro [36]. PFKFB4 generates fructose-2,6-biphosphate which is responsible for the allosteric regulation of PFK1, suggesting that interference with PFK1 activity is detrimental for GBM growth. Nevertheless in our analysis we only identified PFK1, but not PFKFB4 as essential for tumor progression in vivo.

PDK1 knockdown increased mouse survival up to 20.9%. Via inhibition of pyruvate dehydrogenase, PDK1 is a gate-keeping mitochondrial enzyme preventing the entry of pyruvate into the TCA cycle thereby inhibiting the oxidative phosphorylation. Our data supports previous work implicating PDK1 in different cancers and indicating the beneficial effect of its inhibitor DCA in promoting tumor cell apoptosis and inhibiting cancer growth [37, 38]. Recently DCA has also been investigated in the treatment of GBM patients [39], although no large clinical studies are yet available. A recent pre-clinical study provided evidence for a synergistic effect of DCA with bevacizumab albeit this work was carried out in a subcutaneous U87 model [40].

PGAM1 has been reported to be overexpressed in high grade glioma [12]. Here we provide the first indication that PGAM1 plays an essential role in GBM and that PGAM1 silencing in intracranial tumors improves mouse survival. PGAM1 has been identified as a potential therapeutic target in hepatocellular carcinoma [41], however, its implication in cancer cell proliferation remains poorly understood and little is known on its regulation. Similarly, in our study the knockdown of the neuronal and non-neuronal form of enolase (ENO2 and ENO1 respectively) led to a significant survival benefit in mice. Knockdown of ENO2 was previously reported to affect GBM cell migration and sensitize them to hypoxia, radiotherapy and chemotherapy [42]. Of note in our hands ENO1 knockdown was more beneficial for mouse survival than ENO2 knockdown, despite the less efficient gene silencing obtained with ENO1 (S3 Fig).

Finally, we show the importance of HK2 and ALDOA in GBM growth in patient derived GBM xenografts. This is in agreement with previous work showing that HK2 depletion slowed U87 cell growth in vivo and restored oxidative phosphorylation in these cells [43]. Here we show that HK2 depletion in intracranial patient derived GBM xenografts led to a survival improvement of 11.8%. Similar to PDK1, interference with HK2 does not only affect cellular metabolism, but also induces apoptosis thereby conferring a growth advantage to cancer cells [44].

The last part of our study focused on testing chemical compounds known to inhibit glycolysis and shows that chemical interference with PFK1 activity through clotrimazole is effective for GBM treatment. Unfortunately however the effect was less pronounced than with the gene knockdown which is likely due to poor drug potency and limited availability in the tumor. Moreover like most available glycolysis related drugs, clotrimazole is rather unspecific and its mechanism of action is poorly understood. Known as an anti-fungal agent, it has been shown to affect PFK1 activity as well as HK2 binding to the mitochondrial outer membrane [32, 45]. Thus there is a need for more potent and more specific small molecule drugs to target the glycolytic pathway.

In conclusion, using a comprehensive analysis of the glycolytic metabolism in several in vitro and in vivo GBM models, we show that glycolysis is a promising target for GBM therapy, especially though the specific inhibition of the enzymes PFK1 and PDK1. To improve chemical inhibition of these targets, efforts should be concentrated on the synthesis of drugs with high potency and specificity for these two proteins.

## Supporting Information

S1 Fig Induction of glycolysis upon hypoxia. **A**.Differentially expressed genes (DEGs) between hypoxia (12h and 7d separately) and normoxia were determined with ANOVA (FDR<0,001, any fold change) and commonly altered genes were extracted from the Venn diagrams (Fig 1A). 301 and 348 genes were significantly modulated (up or down-regulated) after 12h and 7d respectively under hypoxia in all cell lines analysed. DEG lists were submitted to the DAVID database (DAVID 6.7; http://david.abcc.ncifcrf.gov/) for functional enrichment analysis. Significantly deregulated Gene Ontology (GO) terms after 12h (left) and 7d (right) are presented A strong increase in the concentration of lactate was observed in the extracellular medium of GBM cells subjected to 48h 0.1% O_2_ (* p<0.05, n = 3).
(TIF)

S2 Fig shRNA knockdown efficacy in U87 and NCH644 determined by QPCR. QPCR confirmed the silencing of glycolysis-related genes in shRNA-expressing NCH644 and U87 glioma cells. The residual expression of silenced genes was confirmed for each clone separately and compared to the control clone (n = 3; *** p<0.001; ** p<0.01; p<0.05).
(TIF)

S3 Fig shRNA knockdown efficacy in mouse xenografts determined by QPCR. Total RNA was extracted from tumor mass developed in xenografts during the survival study. The residual expression of silenced genes was confirmed for each clone separately and compared to the control clone. QPCR confirmed the silencing of glycolysis-related genes (n = 5; *** p<0.001; ** p<0.01 * p<0.05).
(TIF)

S1 Table Primers used in the study. (DOCX)

S2 Table Sequence of the 12 shRNAs used for in vitro and in vivo studies. Each shRNA was tagged with a barcode sequence. After tumor resection and DNA extraction, the barcode sequences were amplified and quantified by next generation sequencing (NGS).
(DOCX)

S3 Table Gene expression changes for genes related to glucose metabolism. Genes associated with glycolysis were selected from the Kegg pathway and Gene ontology databases. The pentose phosphate pathway (PPP) genes were depicted from (30). *PDK1* and glucose transporters *SLC2A1* and *SLC2A3* were added manually as genes closely related to glycolysis. Expression of glycolysis-associated genes was analysed within DEG lists for short term hypoxia (12h) and long term hypoxia (7d) versus normoxia. Differentially expressed genes between hypoxic and normoxic cells were determined with ANOVA. Cut-off was set up for FDR<0.001 (Any fold change). Fold changes are presented only for the genes were significantly altered (FDR<0.001).
(DOCX)

S4 Table IC50 on adherent glioma cells. Different glioma cell lines were exposed to the indicated compounds for 72 hours and the cytotoxic effects were assessed using the SRB assay. A concentration gradient of each compound was used for IC_50_ determination in the different cell types. The IC_50_ was determined by the SRB assay on three adherent cell lines U87, U251, T98G. Results are presented as a mean ± SEM of three independent experiments performed in triplicates. N: normoxia; H: hypoxia.
(DOCX)
