# Supplementary figures and images for: Comprehensive Analysis of Glycolytic Enzymes as Therapeutic Targets in the Treatment of Glioblastoma
Source: PLoS One. 2015 May 1;10(5):e0123544. doi: 10.1371/journal.pone.0123544 (PMC4416792; doi:10.1371/journal.pone.0123544)

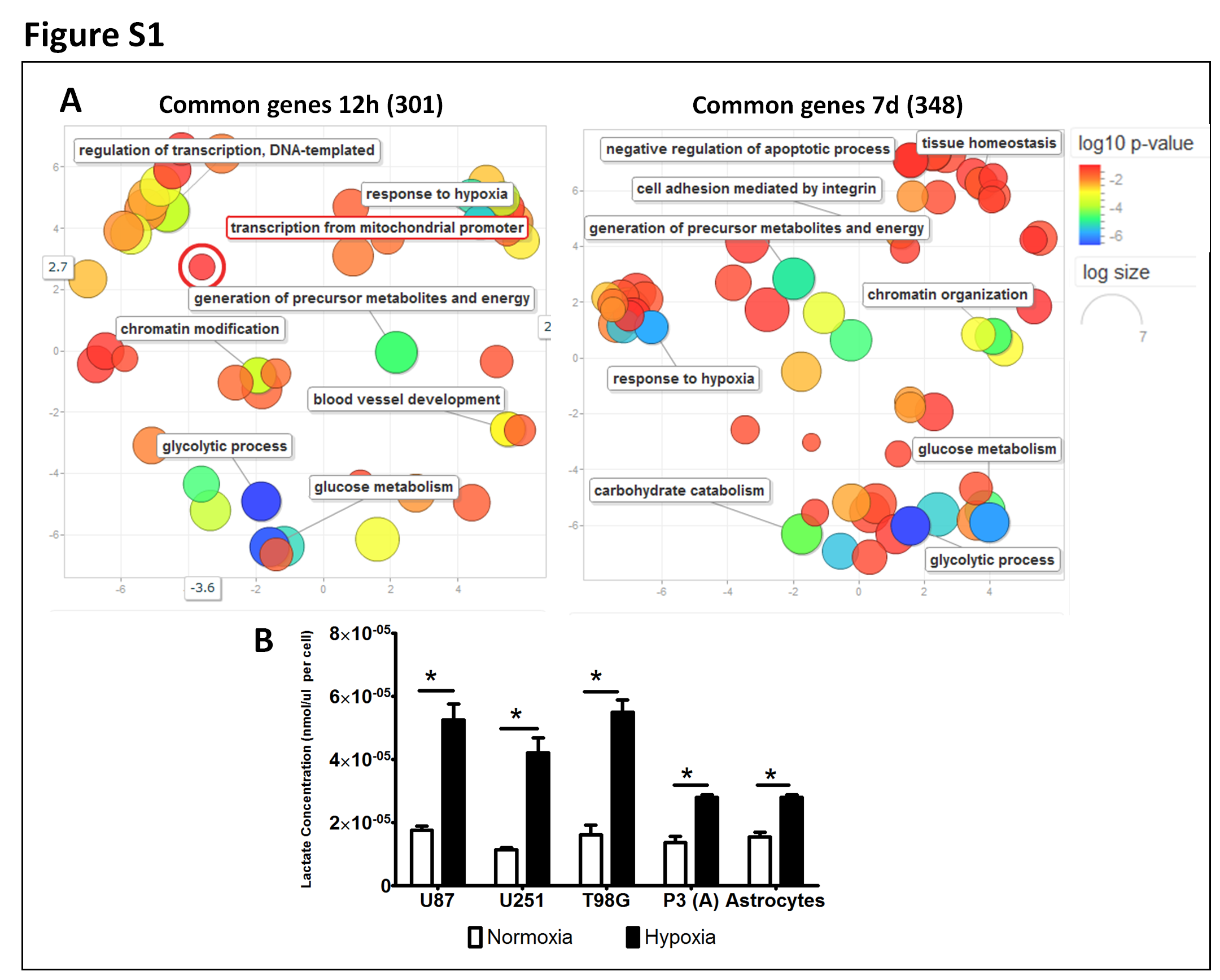

Supplement: S1 Fig — A.Differentially expressed genes (DEGs) between hypoxia (12h and 7d separately) and normoxia were determined with ANOVA (FDR<0,001, any fold change) and commonly altered genes were extracted from the Venn diagrams (Fig 1A). 301 and 348 genes were significantly modulated (up or down-regulated) after 12h and 7d respectively under hypoxia in all cell lines analysed. DEG lists were submitted to the DAVID database (DAVID 6.7; http://david.abcc.ncifcrf.gov/) for functional enrichment analysis. Significantly deregulated Gene Ontology (GO) terms after 12h (left) and 7d (right) are presented A strong increase in the concentration of lactate was observed in the extracellular medium of GBM cells subjected to 48h 0.1% O2 (* p<0.05, n = 3). (TIF) [file pone.0123544.s001.tif]

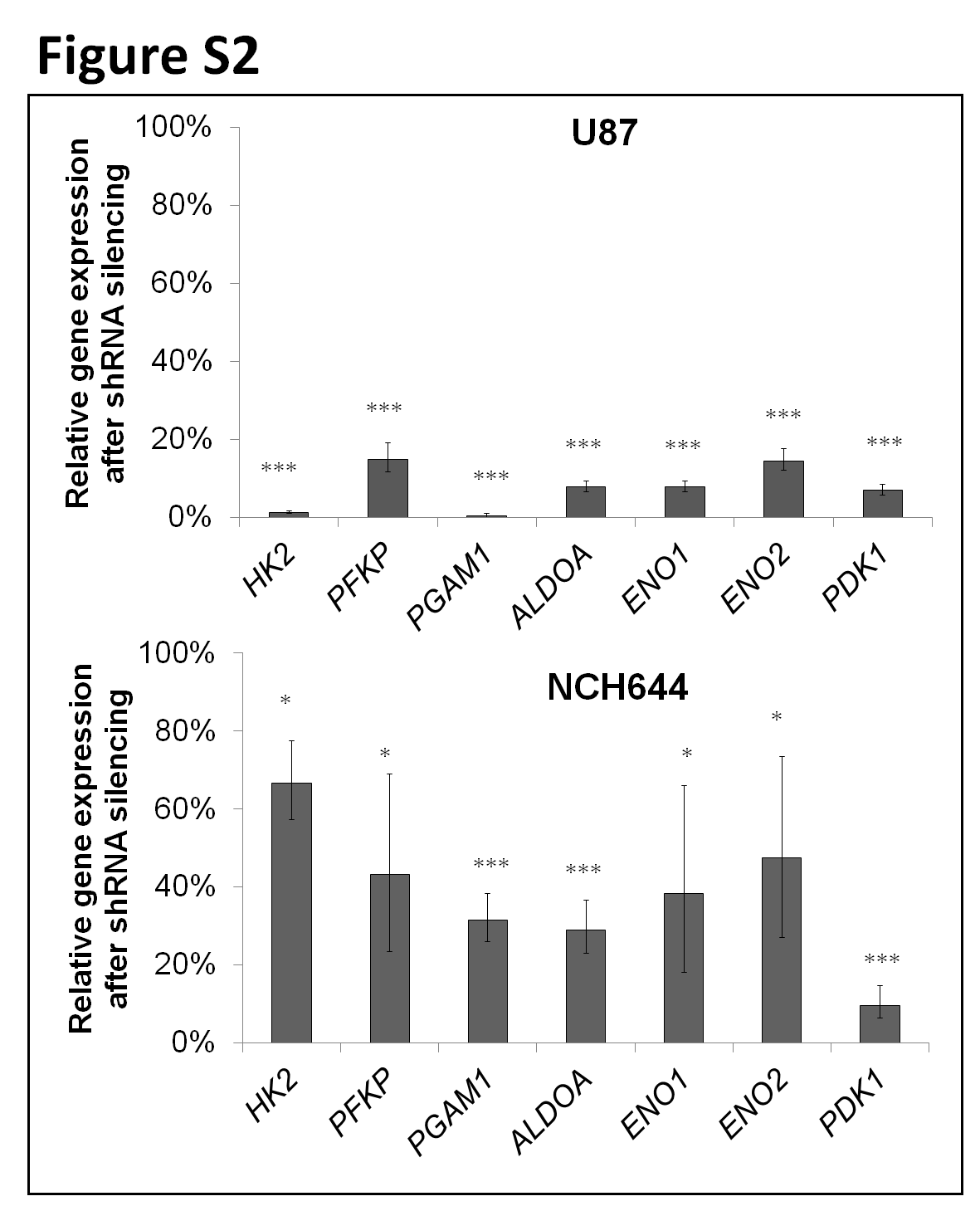

Supplement: S2 Fig — QPCR confirmed the silencing of glycolysis-related genes in shRNA-expressing NCH644 and U87 glioma cells. The residual expression of silenced genes was confirmed for each clone separately and compared to the control clone (n = 3; *** p<0.001; ** p<0.01; p<0.05). (TIF) [file pone.0123544.s002.tif]

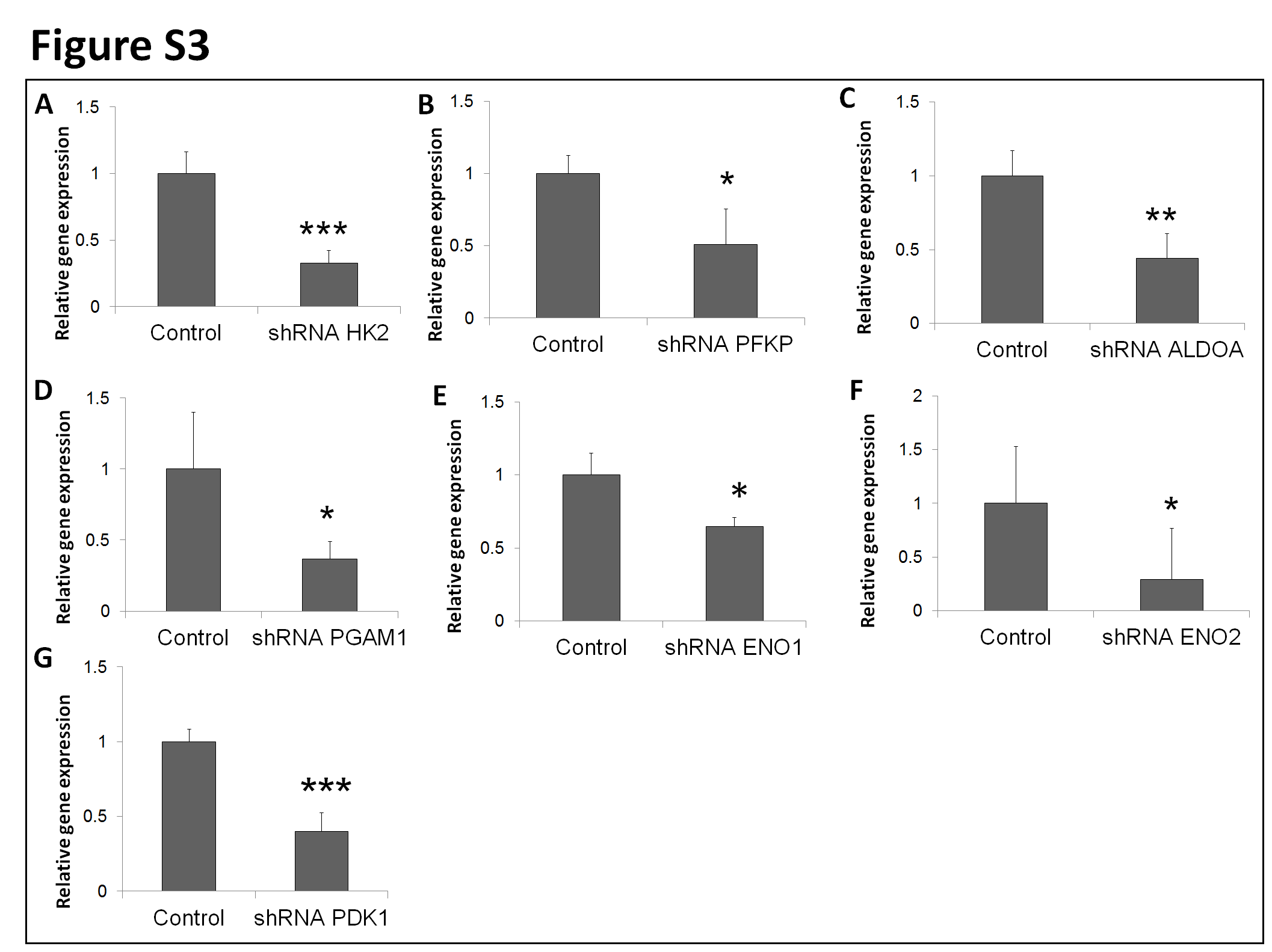

Supplement: S3 Fig — Total RNA was extracted from tumor mass developed in xenografts during the survival study. The residual expression of silenced genes was confirmed for each clone separately and compared to the control clone. QPCR confirmed the silencing of glycolysis-related genes (n = 5; *** p<0.001; ** p<0.01 * p<0.05). (TIF) [file pone.0123544.s003.tif]
