# Supplementary material for: Comprehensive Analysis of Glycolytic Enzymes as Therapeutic Targets in the Treatment of Glioblastoma
Source: PLoS One. 2015 May 1;10(5):e0123544. doi: 10.1371/journal.pone.0123544 (PMC4416792; doi:10.1371/journal.pone.0123544)
Supplement: S1 Table — (DOCX) [file pone.0123544.s004.docx]

**Table S1. Primers used in the study.**

| **Target** | **Primer F** | **Primer R** |
| --- | --- | --- |
| *ACTIN* | CATGTACGTTGCTATCCAGGC | CTCCTTAATGTCACGCACGAT |
| *ALDOA* | TCACCGCATCGTGGCACCTG | GAAGCGCCGGTTCTCCTCGG |
| *ENO1* | TGTGGGTACCCGGAGCACGG | TAGCCACTGGGTCTCGTCGCC |
| *ENO2* | ATCAACTCCACCATCGCGCCA | TCTCAGTCCCATCCAACTCCAGCA |
| *EZRIN* | TGCCCCACGTCTGAGAATC | CGGCGCATATACAACTCATGG |
| *HK2* | TGCCACCAGACTAAACTAGACG | CCCGTGCCCACAATGAGAC |
| *PDK1* | TGGTGGAAAAGGCAAAGGAAGTCCA | ACAGACGCCTAGCATTTTCATAGCCA |
| *PFKP* | GCATGGGTATCTACGTGGGG | CTCTGCGATGTTTGAGCCTC |
| *PGAM1* | AGGTCACTGCCTACTGCCTG | ACATCACCACGCAGGTTACAT |
