# Supplementary material for: Comprehensive Analysis of Glycolytic Enzymes as Therapeutic Targets in the Treatment of Glioblastoma
Source: PLoS One. 2015 May 1;10(5):e0123544. doi: 10.1371/journal.pone.0123544 (PMC4416792; doi:10.1371/journal.pone.0123544)
Supplement: S2 Table — Each shRNA was tagged with a barcode sequence. After tumor resection and DNA extraction, the barcode sequences were amplified and quantified by next generation sequencing (NGS). (DOCX) [file pone.0123544.s005.docx]

**Table S2. Sequence of the 12 shRNAs used for *in vitro* and *in vivo* studies.** Each shRNA was tagged with a barcode sequence. After tumor resection and DNA extraction, the barcode sequences were amplified and quantified by next generation sequencing (NGS).

| **Gene symbol** | **Human Gene Description** | **Barcode sequence** | **Sense shRNA** |
| --- | --- | --- | --- |
| *ALDOA* | aldolase A, fructose-bisphosphate | TAATGCGGCCGGCCAGGCTATTTTTGTTGGTTTCGTGCTTATGTGTGTATCATACTACTTTGGGTTGAGAACGTAGGGC | CCATCAACCTCAATGCCAT |
| **Control** | **Non-silencing-GIPZ lentiviral shRNAmir control** | **GCCATTACTCCGTCTCGTGTCTTGTTGCATATGTCTGCTGGTTTGTTTGATGTTGTTTGC** |  |
| *ENO1* | enolase 1, (alpha) | GTGAGGTCTTTTCTGGACGTGTTGAAGGAAGAAGTGGTTGCCATGCACGTGGACATGGTG | CCCTAGAGCTCCGGGACAA |
| *ENO2* | enolase 2 (gamma, neuronal) | TAATGCGGCCGGCCACTGTTGTCTGTTTTGATCTTCTGTTCGGATGGGTCCTTAGTCGAGTAGGCCGTCTTTGTCGGGC | CGGCCTTCAACGTGATCAA |
| *HK2* | hexokinase 2 | TAATGCGGCCGGCCAGACCACTTTTGCGTGGGCGGGTCTGCTTTTTGGGGTAGGTGGGTGTGCGATTGCTAGCGTGGGC | CTCTTTAACCTCATCTACA |
| *PFKFB4* | 6-phosphofructo-2-kinase/fructose-2,6-biphosphatase 4 | TAATGCGGCCGGCCAAACGTACTAGGTGGATCTTGATATTAGTCTTGTTCTGTCTGTTTTTTATTGTTTTGGGCGGGGC | CAGGCAAATTCTTGCAAAT |
| *PFKP* | phosphofructokinase, platelet | TAATGCGGCCGGCCATTGGCGCGGTACCTGGGCATTTGTTTGGTATTTTTTTTGGTCGTTGCTTGATCTGGTGTGGGGC | GTGTTTGACTGCAGGAAGA |
| *PGAM1* | phosphoglycerate mutase 1 (brain) | TGTTCAAGTAGGTTGAATTTGAGCGAGAGGCGAAGCACTGGCAGCACGGGGGGTGGGAAT | CCTTCTGGAATGAAGAAAT |
| *PGM1* | phosphoglucomutase 1 | TAATGCGGCCGGCCATCTGTGTCTATGCTGTTCTTGTCTTTTGGGTTTTTGGGTGGTGTGTTTCGTTGCATCTGGGC | GGCTGTACATCGATAGCTA |
| *SLC2A1* | solute carrier family 2 (facilitated glucose transporter), member 1 | TAATGCGGCCGGCCAGGCGCGTGGACGTTCTTGGTTGTCGGCTCGGGGTGGGTGTTGTTTTAGTGATTTATGGTGTGGGC | CAGAGAATATATACATTCT |
| *SLC2A3* | solute carrier family 2 (facilitated glucose transporter), member 3 | GGTGTTTATCGACCACACCCCGACTGTGTTCCAGGGAAGTATCGGCGGTGGATAGGCCGA | GGAGAAATGTTGTTCATTA |
| *PDK1* | Pyruvate dehydrogenase kinase I | TAATGCGGCCGGCCATGTTAGCTCGTGTGGTTGTCGCTTGCGTTGGTTCGTATTCGGTTATTAGTCACGGGGTGCGGGC | CTGTCAGACTGGCAAATAT |
