# Supplementary material for: Comprehensive Analysis of Glycolytic Enzymes as Therapeutic Targets in the Treatment of Glioblastoma
Source: PLoS One. 2015 May 1;10(5):e0123544. doi: 10.1371/journal.pone.0123544 (PMC4416792; doi:10.1371/journal.pone.0123544)
Supplement: S3 Table — Genes associated with glycolysis were selected from the Kegg pathway and Gene ontology databases. The pentose phosphate pathway (PPP) genes were depicted from (30). PDK1 and glucose transporters SLC2A1 and SLC2A3 were added manually as genes closely related to glycolysis. Expression of glycolysis-associated genes was analysed within DEG lists for short term hypoxia (12h) and long term hypoxia (7d) versus normoxia. Differentially expressed genes between hypoxic and normoxic cells were determined with ANOVA. Cut-off was set up for FDR<0.001 (Any fold change). Fold changes are presented only for the genes were significantly altered (FDR<0.001). (DOCX) [file pone.0123544.s006.docx]

**Table S3. Gene expression changes for genes related to glucose metabolism.** Genes associated with glycolysis were selected from the Kegg pathway and Gene ontology databases. The pentose phosphate pathway (PPP) genes were depicted from [[1](#_ENREF_1)]. *PDK1* and glucose transporters *SLC2A1* and *SLC2A3* were added manually as genes closely related to glycolysis. Expression of glycolysis-associated genes was analysed within DEG lists for short term hypoxia (12h) and long term hypoxia (7d) versus normoxia. Differentially expressed genes between hypoxic and normoxic cells were determined with ANOVA. Cut-off was set up for FDR<0.001 (Any fold change). Fold changes are presented only for the genes were significantly altered (FDR< 0.001).

* The pentose phosphate pathway (PPP)- related genes

Genes chosen for the in vitro and in vivo knockdown experiments are in **bold**

|  | **NCH421k** | | **NCH644** | | **U87** | | **U251** | |
| --- | --- | --- | --- | --- | --- | --- | --- | --- |
| Hypoxia | 12h | 7d | 12h | 7d | 12h | 7d | 12h | 7d |
| ACSS1 | -1.53474 | -2.12532 |  | -1.8134 |  |  |  |  |
| ACSS2 |  |  | 7.95929 |  |  | -2.11341 |  |  |
| ADH1A |  |  |  |  |  |  |  |  |
| ADH1B |  |  |  |  |  |  |  |  |
| ADH1C |  |  |  |  |  |  |  |  |
| ADH4 |  |  |  |  |  |  |  |  |
| ADH5 |  |  |  |  |  |  |  |  |
| ADH6 |  |  |  |  |  |  |  |  |
| ADH7 |  |  |  |  |  |  |  |  |
| ADPGK |  |  |  |  |  | 1.43683 |  | 1.47391 |
| AKR1A1 |  |  |  |  |  | -1.49817 |  |  |
| ALDH1A1 |  |  |  |  |  |  |  |  |
| ALDH1B1 | -1.76988 | -1.49961 | -1.8792 | -1.8792 | -3.22597 | -2.9266 |  |  |
| ALDH2 |  |  |  |  |  |  |  |  |
| ALDH3A1 |  |  |  |  |  |  |  | -2.9697 |
| ALDH3A2 |  |  |  |  | -1.35445 | -1.71831 |  | -1.7637 |
| ALDH3B1 |  |  |  |  |  | 1.65196 |  |  |
| ALDH3B2 |  |  |  |  |  |  |  |  |
| ALDH7A1 |  | -1.9356 | 1.59749 |  |  | -1.69424 |  |  |
| ALDH9A1 |  | -1.42459 |  |  |  |  |  |  |
| **ALDOA** | 1.46554 | 1.47932 | 1.6179 | 1.43752 |  |  |  | 1.3658 |
| ALDOB |  |  |  |  |  |  |  |  |
| ALDOC | 4.22785 | 4.53783 | 7.78982 | 4.11718 | 8.65455 | 7.7862 |  | 2.77664 |
| BPGM |  |  | -1.48966 |  | -1.34324 | -2.16287 |  |  |
| DLAT | -1.85666 | -1.96935 |  | -1.44387 |  | -3.62745 |  |  |
| DLD |  | -1.38424 |  | -1.48387 |  | -1.7554 |  | -1.47738 |
| **ENO1** |  |  | 1.44782 | 1.34263 |  | 1.39833 |  |  |
| **ENO2** | 4.3831 | 5.3341 | 6.31245 | 7.64352 | 3.15836 | 4.13418 | 4.56236 | 4.6429 |
| ENO3 |  |  |  | 1.6999 |  | 1.46133 | 1.57427 | 2.63381 |
| ENO4 |  |  |  |  |  |  |  |  |
| FBP1 |  |  |  |  |  |  |  | 1.4662 |
| FBP2 |  |  |  |  |  |  |  |  |
| G6PC |  |  |  |  |  |  |  |  |
| G6PC2 |  |  |  |  |  |  |  |  |
| G6PC3 |  |  |  |  |  |  |  |  |
| GALM |  |  |  |  | 1.56189 |  | 1.6165 |  |
| GAPDH |  |  |  |  |  |  |  |  |
| GAPDHS |  |  |  |  |  |  |  |  |
| GCK |  |  |  |  |  |  |  |  |
| GPI | 1.85542 | 1.83199 | 1.7296 | 1.75239 | 1.4192 | 2.4986 | 1.6291 | 1.34761 |
| HK1 | 2.5471 | 1.91289 | 1.8613 | 1.58696 | 1.34321 |  | 1.52112 |  |
| **HK2** | 3.84315 | 3.77368 | 2.3242 | 3.2727 | 5.25441 | 5.98321 | 4.3697 | 3.79183 |
| HK3 |  |  |  |  |  |  |  |  |
| HKDC1 |  |  |  |  |  |  |  |  |
| LDHA | 1.35454 |  | 1.4848 |  |  |  |  | 1.58696 |
| LDHAL6A |  |  |  |  |  |  |  |  |
| LDHAL6B |  |  |  |  |  |  |  |  |
| LDHB |  | -1.46547 |  | -1.45764 |  | -2.83646 |  | -1.38491 |
| LDHC |  |  |  |  |  |  |  |  |
| MINPP1 | -1.57574 |  |  |  |  |  |  | -1.51924 |
| PCK1 |  |  |  |  |  |  |  |  |
| PCK2 |  |  |  |  |  |  |  |  |
| PDHA1 | -1.316 | -1.38749 | -1.3968 | -2.338 |  | -1.7516 | 1.29428 |  |
| PDHA2 |  |  |  |  |  |  |  |  |
| PDHB |  | -1.38971 |  |  |  |  |  |  |
| **PDK1** | 2.98639 | 2.47562 | 5.71344 | 4.78455 | 2.86756 | 3.33761 | 3.85584 | 4.4511 |
| PDP2 |  |  |  | -1.6823 |  | -5.26463 |  |  |
| PFKFB1 |  |  | 1.29782 |  |  |  |  |  |
| PFKFB2 |  |  | -1.628 |  |  |  |  |  |
| PFKFB3 |  |  |  |  | 3.7834 | 4.6425 | 5.28697 | 2.643 |
| **PFKFB4** | 7.49919 | 7.32542 | 14.9445 | 1.3857 | 5.17766 | 6.67118 | 11.6219 | 6.87848 |
| PFKL | 1.8166 | 1.48536 | 2.63462 | 1.74421 | 1.4425 | 2.83852 | 1.767 | 1.74688 |
| PFKM |  |  |  |  |  |  |  |  |
| **PFKP** | 2.83555 | 2.72325 | 2.36317 | 2.23539 | 1.6496 | 2.15974 | 1.96954 |  |
| **PGAM1** | 1.5167 | 1.4889 | 1.58911 | 1.44214 | 1.45867 | 1.3551 | 1.53496 | 1.37899 |
| PGAM2 |  | 1.6249 |  |  |  |  |  |  |
| PGAM4 | 1.5799 | 1.42323 | 1.47314 | 1.36219 | 1.35531 | 1.22211 | 1.4535 |  |
| PGD* |  | -1.59793 |  |  |  | -1.77913 |  |  |
| PGK1 | 1.71778 | 1.75919 | 1.9156 | 1.8232 | 1.65566 | 2.4874 | 1.583 | 1.63738 |
| PGK2 |  |  |  |  |  |  |  |  |
| PGLS* |  | -1.96576 |  |  |  |  |  |  |
| **PGM1** | 1.4354 | 1.5291 | 2.1581 | 1.65781 |  | 1.63225 | 1.84719 | 1.9118 |
| PGM2 | 1.71361 |  |  | -1.46297 |  |  |  |  |
| PKLR |  |  |  |  |  |  |  |  |
| PKM | 1.4329 | 1.58299 | 1.78167 | 1.46683 | 1.1922 | 1.27222 | 1.21946 |  |
| **SLC2A1** | 3.58988 | 3.5285 | 2.46965 | 2.6227 | 6.35395 | 9.88234 | 1.556 |  |
| **SLC2A3** | 8.3781 | 7.83425 | 9.92622 | 1.873 | 3.68881 | 2.47835 | 9.74714 | 5.9675 |
| TALDO1* |  | -1.84697 |  |  |  | -1.9636 |  | -1.35539 |
| TKT* |  |  |  |  |  | -1.9856 |  | -2.4448 |
| TPI1 | 1.656 | 1.5567 | 1.81574 |  |  | 1.43967 | 1.58881 |  |

1. Kathagen A, Schulte A, Balcke G, Phillips HS, Martens T, et al. (2013) Hypoxia and oxygenation induce a metabolic switch between pentose phosphate pathway and glycolysis in glioma stem-like cells. Acta Neuropathol 126: 763-780.
