# Supplementary material for: Comprehensive Analysis of Glycolytic Enzymes as Therapeutic Targets in the Treatment of Glioblastoma
Source: PLoS One. 2015 May 1;10(5):e0123544. doi: 10.1371/journal.pone.0123544 (PMC4416792; doi:10.1371/journal.pone.0123544)
Supplement: S4 Table — Different glioma cell lines were exposed to the indicated compounds for 72 hours and the cytotoxic effects were assessed using the SRB assay. A concentration gradient of each compound was used for IC50 determination in the different cell types. The IC50 was determined by the SRB assay on three adherent cell lines U87, U251, T98G. Results are presented as a mean ± SEM of three independent experiments performed in triplicates. N: normoxia; H: hypoxia. (DOCX) [file pone.0123544.s007.docx]

**Table S6. IC50 on adherent glioma cells.** Different glioma cell lines were exposed to the indicated compounds for 72 hours and the cytotoxic effects were assessed using the SRB assay. A concentration gradient of each compound was used for IC_50_ determination in the different cell types. The IC_50_ was determined by the SRB assay on three adherent cell lines U87, U251, T98G. Results are presented as a mean ± SEM of three independent experiments performed in triplicates. N: normoxia; H: hypoxia.

| **Cell line** | **U87** | | **U251** | | **T98G** | |
| --- | --- | --- | --- | --- | --- | --- |
| Compound/Condition | N | H | N | H | N | H |
| Clotrimazole (μM) | 25.5±7 | 57.1±6 | 9.11±0.5 | 46.5±1.3 | 21.1±5 | 56.6±8.7 |
| Citrate (mM) | 19±8 | 6±2 | No effect | No effect | 42±12 | 81±19 |
| Dichloroacetate (mM) | 36±5 | 116±8 | 23.1±3 | 400±6.5 | 29.5±3.1 | 106±11 |
| Bromopyruvate (μM) | 236±4 | 140±5 | 97±8 | No effect | 377±7.1 | 452±10.4 |
